# Impact of soil fissure status on microbial community in mining-disturbed area, the northern Shaanxi province

**DOI:** 10.3389/fmicb.2024.1463665

**Published:** 2024-08-29

**Authors:** Liang Guo, Xianglong Chen, Yizhi Sheng, Nuan Yang, Enke Hou, Haisong Fang

**Affiliations:** ^1^College of Geology and Environment, Xi’an University of Science and Technology, Xi’an, China; ^2^State Key Laboratory of Biogeology and Environmental Geology & MOE Key Laboratory of Groundwater Circulation and Environment Evolution, China University of Geosciences, Beijing, China

**Keywords:** microbial community, soil biodiversity, mining disturbance, soil fissure, co-occurrence network

## Abstract

Mining disturbance has great impacts on soil physicochemical factors, causing notable differences between pre-mining and after-mining conditions, and between coal mining areas and non-mined areas. However, little is known about whether the fissure statuses induced by mining activities affect the edaphic factors and how soil microbial communities respond to these fissure development states. In this study, we systematically investigated the edaphic factors and microbial communities in a mining disturbance area exhibiting the full development status of soil fissures, where the sampling sites were divided into soil fissure development and closure zones. Microbial alpha-and beta-diversity, correlation coefficient matrix, non-metric multi-dimensional scaling, principal co-ordinates analysis, mantel test, and microbial co-occurrence network were employed to elucidate variations, correlations, and interactions between edaphic factors and microbial communities under the two different soil fissure states. Results suggested that soil physicochemical properties were significantly affected by fissure states, showing an increasing trend in soil moisture content and soil nutrients. The associations among edaphic factors have weakened during the soil fissure development process. Soil microbial communities showed different compositions and the underlying influential mechanisms between two soil fissure states. Soil moisture content, pH, particle compositions, organic matter, and heavy metals largely affected microbial communities. Rare species were vulnerable to mining disturbance and were keystone taxa that reinforced the overall interconnections of the soil microbial community (e.g., *Nordella*, *Sphingomonas*, *Massilia*, and *Rubritepida*). Our study revealed the impacts of distinct fissure states on the soil physicochemical properties and microbial communities, and the edaphic conditions showed key contributions to the soil microbial communities, particularly the abundance and ecological roles of rare species.

## 1 Introduction

China’s energy supply heavily relies on coal resources, although vigorously advocating the “two-carbon policy” in recent years. The Ordos Plateau and its adjacent areas had been bearing tremendous shallow-buried coal deposits and became one of the most important coal-producing areas in China. However, the development of the coal mining industry has resulted in soil fissures, land subsidence, degradation, etc. in mining disturbance areas (Liu et al., 2015; Wang G. et al., 2017; Yang et al., 2019), and further causing countless losses, severe anthropogenic geological disasters. Mining fissures are categorized into three types: tensile fissures, collapse fissures, and sliding fissures. The dynamic characteristics of mining-induced fissures undergo a development cycle of fissure-expansion-closure (Liu et al., 2019; Kang et al., 2023). Ground fissures showed an inverse C-shape feature and developed in advance of the coal seam working face excavation, and then distributed in the “O” form on the boundary of the working face after subsidence stabilized (Hou et al., 2022).

The emergence of soil fissures has a profound influence on the ecological environment and soil physicochemical factors (Liu et al., 2019). Mining activities significantly altered soil organic matter (SOM), total nitrogen (TN), and available phosphorus (AP). Upon mining disturbances, SOM, TN, and AP typically decreased by 35.49, 23.56, and 38.06%, respectively, compared to the pre-mining topsoil (Wang et al., 2021). Other lowered indicators included soil moisture content, soil clay content, and available potassium (Luo et al., 2019; Ma et al., 2019). Moreover, in the vertical profile of the mining disturbance area, most soil parameters (e.g., SOM, AP) were lower than those in the nonmined area at all depths. In contrast, these indicators showed an opposite trend in the nonmined area with increasing depth, indicating that mining activities altered the vertical distribution of edaphic factors (Shi et al., 2017). However, in other cases, soil nutrients (e.g., TP, TN) showed an increasing trend with the development of soil fissures (Peng et al., 2020; Long et al., 2022).

Soil microorganisms are ubiquitously present in soil and have tight correlations with their environment and nutrient levels (Chapman et al., 2018). Therefore, soil microbial communities are highly susceptible to the changing environment in mining disturbance areas. Compared to non-mined sites, soil microbial community structure and diversity in the coal mining area would be impaired and significantly influenced by soil properties (Ezeokoli et al., 2019; Yuan et al., 2022). Under the mining disturbance conditions, soil pH was deemed as one of the most influential factors for shaping microbial diversity, taxonomic composition, and ecological distribution, especially along the mining disturbance gradient (Xiao et al., 2021a). Additionally, other factors influencing soil bacterial community included conductivity (EC), soil moisture content, soil depth, nutrient concentrations, etc. (Banning et al., 2012; Guo et al., 2021; Sheng et al., 2021b). Mining activities have been shown to reduce soil microbial abundance and diversity (Shi et al., 2017). There were also concerns regarding the recovery of the soil environment and microbial communities after mining activity ceased and the duration required for recovery. While soil pH, moisture content, nutrients, and heavy metals were stabilized and reconnected with microbial communities after soil restoration, no matter the positive or negative restoration processes, these attributes rarely return to their original levels, even after decades of restoration efforts (Banning et al., 2012; de Quadros et al., 2016).

In all, there remains a substantial knowledge gap regarding whether the different fissure states induced by mining activities have an impact on soil microbial communities, and how the edaphic factors and microbial communities co-varied during the different fissure development states. Therefore, based on field investigation findings, we selected a shallow-buried coal seam located in the Shaanxi Province, known for its extensively developed soil fissures. An integrated approach combining soil edaphic and microbial community analysis was employed to elucidate soil physicochemical variables and microbial community variation, as well as the correlation between the soil edaphic factors and microbial communities under different soil fissure states. Soil physicochemical properties, including size distribution of soil particles, inorganic variables, organic components, and heavy metals, were determined to decipher soil edaphic conditions in mining disturbance areas. Soil bacteria and archaea communities were analyzed using high-throughput sequencing of the 16S rRNA gene. This study aimed to uncover the variations and interconnections of the edaphic condition and microbial community across distinct soil fissure states.

## 2 Materials and methods

### 2.1 Site description

The study area is located at the junction of Shaanxi and Inner Mongolia Provinces, northeast of the Yushenfu mining area, one of the most coal production fields in China. This area is characterized by a temperate semi-arid continental monsoon climate with dryness, low precipitations, and meteorological disasters. The annual temperature is approximately 6.6°C, while the annual precipitation ranges from 109 mm to 819 mm, and 76% of total precipitation is concentrated from June to September (Xiao et al., 2020). In contrast, the annual evaporation is seven times greater than the rainfall. This vast deficit between precipitation and evaporation has led to the scarcity of regional water resources and fragile ecological environments. Soil texture is dominated by aeolian sandy soil and loess, characterized by poor structure, low fertility, and low resistance to erosion (Hou et al., 2022). Therefore, surface soil is sensitive to mining disturbance, and fissures are extensively developed. The sampling site was chosen at one of the most productive coal mines, the Ningtiaota coalfield, with flat topography in the Yushenfu mining area (Huang et al., 2018). In this area, the active mining depth of the Ningtiaota coalfield is less than 200 m, causing surface fissures to reach the topsoil directly. Due to shallow-buried coal seams and large coal yields, two different types of soil fissures developed: fissure development state and fissure closure state (Hou et al., 2022).

### 2.2 Sampling method and soil edaphic properties measurement

**Figure 1:**
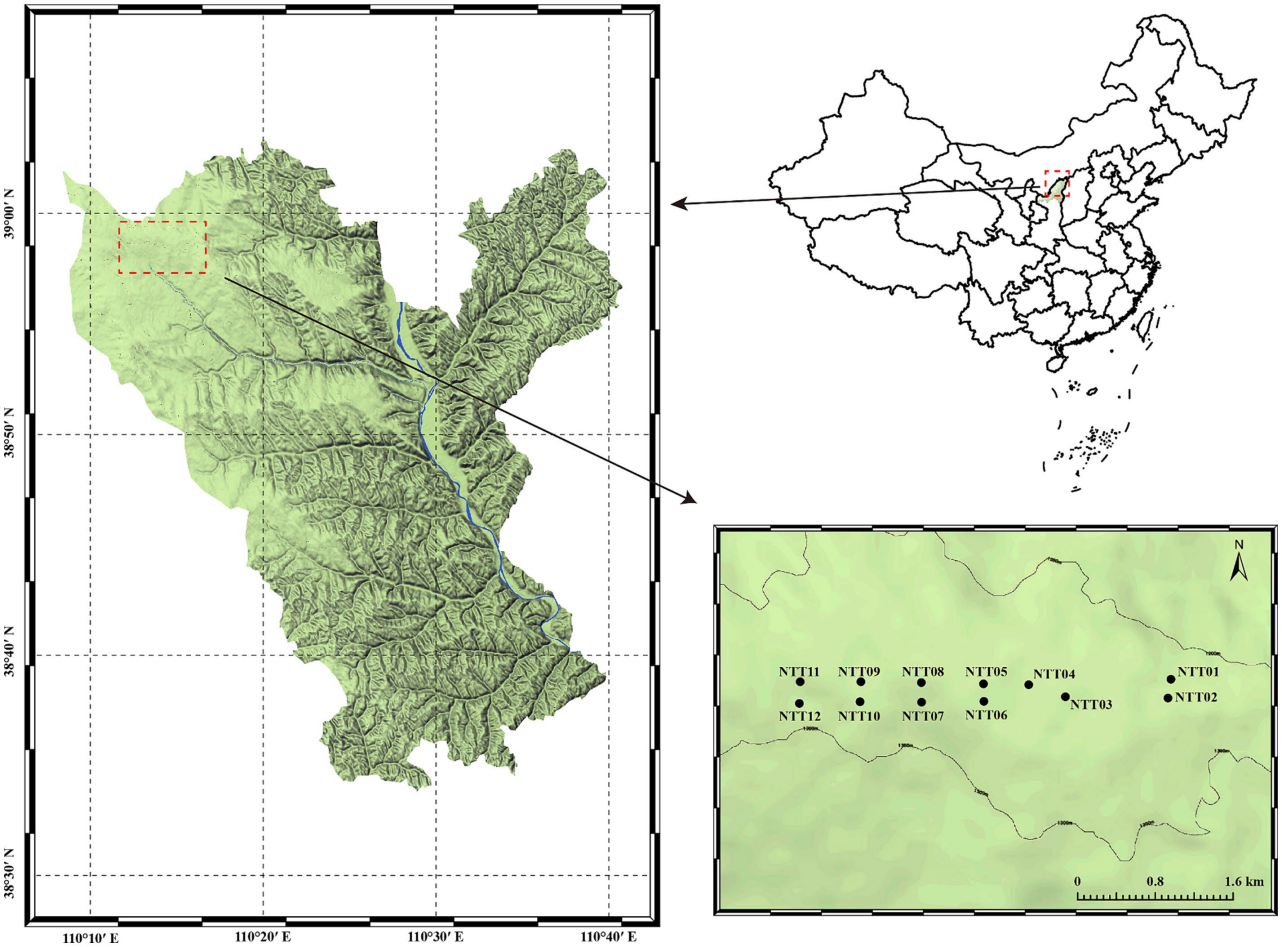
Simplified topographical map of the study area and sampling locations.

Soil sampling sites were strategically chosen based on the field investigations during September 2021, with sampling points distributed across the soil fissure development and closure areas of the coal mine (Figure 1; Supplementary Figure S1). A total of 12 sampling points were selected based on the soil fissure states, of which half were distributed at the soil fissure development zone (FDZ, including NTT02, NTT03, NTT06, NTT07, NTT10, and NTT12), and the other half were distributed at the soil fissure closure zone (FCZ, including NTT01, NTT04, NTT05, NTT08, NTT09, and NTT11). Soil samples were collected 10 cm below the surface to avoid interference of potential surface pollutants. For each sample, five replicates were uniformly collected within a 1 m radius and mixed to effectively form one soil sample based on the five-point sampling method (Deng et al., 2013). Each collected sample was split into two parts for physicochemical and biological analysis. For the edaphic factors determination, rocks and roots were removed from the soil samples, which were then air-dried and sieved through a 0.25 mm sieve before the measurements (Xin et al., 2016). Soil samples for microbial analysis were collected by sterile shovel, stored in dry ice immediately in the field, and transported into the laboratory within 48 days for further DNA extraction (Sheng et al., 2021b). All samples were preserved under 4°C before being transported to the laboratory within 48 h.

Soil samples were analyzed for a variety of geochemical parameters. Soil pH and electrical conductivity (EC) were tested using a glass electrode in water (1:2.5 and 1:5 soil/water ratio, respectively). Soil texture, soil moisture content (SMC), cation exchange capacity (CEC), total phosphorus (TP), available phosphorus (AP), total potassium (TK), and available potassium (AK) were determined by standard methods (Lu, 1999). The soil organic carbon (SOC) content was measured using the dichromate oxidation method (Kalembasa and Jenkinson, 1973). Total carbon (TC) and dissolved organic carbon (DOC) content were measured by the total organic carbon analyzer (TOC-V Series SSM-5000A). Soil microbial biomass carbon (MBC) and nitrogen (MBN) were determined by the chloroform fumigation-extraction method, which is the most common technique for measuring microbial biomass elements (Cleveland and Liptzin, 2007). Heavy metals, including cadmium (Cd), iron (Fe), copper (Cu), manganese (Mn), and chromium (Cr), were estimated by atomic absorption spectrometry (McLaughlin et al., 2000; Tüzen, 2003). Hydride generation atomic fluorescence detection measured total arsenic (As) in soil samples (Shi et al., 2003). Total nitrogen (TN) was measured by the Kjeldahl method, and ammonium nitrogen (NH_4_-N) and nitrate nitrogen (NO_3_-N) were determined by extracting fresh soil with 1 mol/L KCl for 30 min and were analyzed using an autoanalyzer. Alkali-hydrolyzable nitrogen (AHN) was regarded as soil-available nitrogen and determined by the diffusion-absorption method (Zhang et al., 2018; Xiao et al., 2019). Nitrite nitrogen (NO_2_-N) was analyzed colorimetrically by extraction method with potassium chloride (KCl, 1 mol/L) solution (Dai et al., 2020). Humus substance (Humus) and its fractions [humin (Hm), humic acid (HA), and fulvic acid (FA)] were determined based on the standard protocols after extraction with a mix of sodium hydroxide (0.1 mol/L) and sodium pyrophosphate (0.1 mol/L) (Sheng et al., 2021b).

### 2.3 Soil DNA extraction and microbial high-throughput sequencing

Soil genomic DNA was extracted using the FastDNA Spin Kit (MP Biomedicals^™^, Fisher Scientific, United States) according to the manufacturer’s instructions. Amplification of partial hypervariable 16S rRNA gene was performed by using bacterial primer pairs 338F (5′-ACTCCTACGGGAGGCAGCAG-3′)/806R (5′-GGACTACHVGGGTWTCTAAT-3′) for V3–V4 region (Liu et al., 2020), and archaeal primer pairs 524F10extF (5′-TGYCAGCCGCCGCGGTAA-3′)/Arch958RmodR (5′-YCCGGCGTTGAVTCCAATT-3′) for V4–V5 region (Liu et al., 2016). Bacterial and archaeal soil genomic DNA was amplified by PCR which consisted of an initial denaturation step (94°C) for 5 min, followed by 30 cycles of 94°C (1 min), 64°C (1 min), and 72°C (1 min), and a final 10 min elongation (72°C) (Sheng et al., 2021b). After PCR, 16S rRNA amplicons were extracted from 2% agarose gels and purified using the AxyPrep DNA Gel Extraction Kit (Axygen Biosciences, United States) and quantified by a fluorescent quantitation system (QuantiFluor^™^-ST, Promega, United States) according to the manufacturer’s instructions. Purified amplicons were pooled in equimolar and paired-end sequenced on an Illumina MiSeq platform. The raw reads were deposited into the NCBI Sequence Read Archive (SRA) database (Accession Number: SRP503985).

### 2.4 Data statistics and bioinformatics analysis

Totally 542,868 bacterial raw reads and 684,944 archaeal raw reads were produced for all samples, and the rarefaction curve showed that the numbers of observed operational taxonomic units (OTU) were close to saturation, indicating a sufficient sequencing depth (Sheng et al., 2016). The sequencing quality controlling of raw reads was conducted by removing low-quality reads using the QIIME pipeline as previously described (Caporaso et al., 2010; Guo et al., 2022). Operational taxonomic units (OTUs) were clustered using UPARSE based on the 97% similarity criteria (Edgar, 2013). The taxonomy of each OTUs was analyzed according to the SILVA (SSU115) database (Wang et al., 2007). Representative OTUs were also blasted against the NCBI taxonomic database^1^ to acquire a more reliable taxonomic resolution for further analysis. Alpha-and beta-microbial diversity were calculated within QIIME, and microbial beta-diversity was conducted by Bray–Curtis metrics based on pairwise distance values (Guo et al., 2022).

The correlation between edaphic factors was measured by the Pearson correlation coefficient. Mantel test was used to evaluate the correlation between soil physicochemical properties and microbial communities. To avoid the bias induced by different units of edaphic factors, soil datasets were scaled before calculating. Pearson correlation analysis and Mantel test were performed and visualized by using “vegan,” and “LinkET” packages in R. Non-metric multi-dimensional scaling (NMDS) and principal co-ordinates analysis (PCoA) were conducted to evaluate the similarity of microbial communities in soil samples. Other statistical analyses, e.g., one-way analysis of variance (ANOVA), and analysis of similarity (ANOSIM) with 9,999 permutations were conducted by using SPSS 19.0 (IBM Company, 2010). Redundancy analysis (RDA, lengths of gradient ≤3.0) was implemented with the forward selection of variables in Canoco 5.0, and the contribution of each edaphic factor to microbial beta diversity and associated statistical tests were calculated. To estimate microbial species co-occurrence patterns, distance-based microbial networks were constructed with those microbial taxa (OTU level) with >50% presence at each sample by the Spearman correlation matrix of pairwise associations among the OTUs (Sheng et al., 2021c). The network topological indices [e.g., nodes, links, average degree (avgK), average clustering coefficient (avgCC), average path distance (GD), within-module connectivity (Zi), and among-module connectivity (Pi)] were calculated to describe the properties of the networks and the network was visualized by the Gephi 0.9.2 as previously described (Deng et al., 2012).

## 3 Results

### 3.1 Impact of fissure status on soil physicochemical properties

The size distribution of soil particles in the study area predominantly consisted of fine sand and silt, with relatively low proportions of clay and coarse sand (Table 1). The content of fine sand in the fissure closure zone (FCZ, 64.8 ± 3.0%) was slightly lower than that in the fissure development zone (FDZ, 67.9 ± 4.0%). In contrast, the contents of silt and clay in the FCZ group were higher than those in FDZ, with silt at 21.85 ± 2.24% and clay at 12.0 ± 0.7% % in FCZ, compared to 20.43 ± 2.9% and 10.2 ± 1.4% in FDZ. Coarse sand, the largest particle in soil, was less than 2% in both FCZ and FDZ groups. Soil pH exhibited minor variations, ranging from 7.95 to 9.05 with an average of 8.6, indicating an alkaline nature. EC and SMC exhibited significant variations. The FCZ group displayed lower EC (41.1 ± 5.6 μs/cm) and SMC (4.77 ± 0.26%) compared to the FDZ group (53.2 ± 3.6 μs/cm and 5.71 ± 0.22%, respectively).

**Table 1:** Soil edaphic factors between soil fissure development and closure zone.

Variables	Unit	FCZ	FDZ	Variance
Clay	<0.002 mm	12.02 ± 0.69	10.19 ± 1.36	17.99%
Silt	0.002–0.02 mm	21.85 ± 2.24	20.43 ± 2.9	6.93%
Fine sand	0.02–0.2 mm	64.79 ± 3.02	67.93 ± 4.0	−4.62%
Coarse sand	0.2–2 mm	1.34 ± 0.43	1.45 ± 0.35	−7.55%
pH		8.50 ± 0.17	8.73 ± 0.08	−2.69%
Electrical conductivity (EC)	μs/cm	41.1 ± 5.62	53.23 ± 3.63	−22.79%
Soil moisture content (SMC)	%	4.78 ± 0.26	5.71 ± 0.22	−16.43%
Cation exchange capacity (CEC)	cmol/kg	5.17 ± 0.56	5.60 ± 0.38	−7.52%
Total nitrogen (TN)	g/kg	0.39 ± 0.05	0.42 ± 0.02	−4.91%
Microbial biomass nitrogen (MBN)	mg/kg	5.55 ± 0.67	8.76 ± 1.32	−36.71%
Nitrate nitrogen (NO_3_-N)	mg/kg	2.36 ± 0.28	4.65 ± 0.41	−49.21%
Ammonium nitrogen (NH_4_-N)	mg/kg	0.49 ± 0.05	0.52 ± 0.06	−6.29%
Nitrite nitrogen (NO_2_-N)	mg/kg	0.03 ± 0.004	0.03 ± 0.001	9.30%
Alkali-hydrolyzable nitrogen (AHN)	mg/kg	23.92 ± 1.84	28.88 ± 1.33	−17.19%
Total carbon (TC)	g/kg	17.34 ± 1.44	22.32 ± 1.13	−22.28%
Microbial biomass carbon (MBC)	mg/kg	53.40 ± 4.84	80.50 ± 9.76	−33.67%
Dissolved organic carbon (DOC)	mg/kg	55.96 ± 2.81	67.51 ± 1.64	−17.11%
Soil organic carbon (SOC)	g/kg	4.33 ± 0.59	4.46 ± 0.28	−2.94%
Humus substance (Humus)	g/kg	1.01 ± 0.1	1.15 ± 0.06	−11.76%
Humic acid (HA)	g/kg	0.68 ± 0.08	0.85 ± 0.09	−20.69%
Humin (Hm)	g/kg	3.31 ± 0.49	3.31 ± 0.24	0.12%
Fulvic acid (FA)	g/kg	0.33 ± 0.03	0.29 ± 0.06	14.27%
Copper (Cu)	mg/kg	5.06 ± 0.55	5.33 ± 0.45	−5.06%
Manganese (Mn)	g/kg	0.25 ± 0.02	0.23 ± 0.01	6.18%
Cadmium (Cd)	mg/kg	0.11 ± 0.02	0.08 ± 0.02	36.50%
Chromium (Cr)	mg/kg	20.62 ± 1.67	17.35 ± 1.35	18.84%
Iron (Fe)	g/kg	22.38 ± 1.51	20.12 ± 1.05	11.22%
Arsenic (As)	mg/kg	5.33 ± 0.67	5.57 ± 0.38	−4.33%
Total phosphorus (TP)	g/kg	0.32 ± 0.02	0.35 ± 0.02	−8.39%
Total potassium (TK)	g/kg	20.43 ± 0.21	20.51 ± 0.22	−0.40%
Available phosphorus (AP)	mg/kg	0.98 ± 0.17	1.27 ± 0.24	−22.71%
Available potassium (AK)	mg/kg	58.5 ± 5.04	72.67 ± 7.36	−19.50%

Statistical value “variance” represented the changes of soil physicochemical variables in the soil fissure closure zone (FCZ) in relative to the soil fissure development zone (FDZ). The reference value referred to the edaphic factors in FDZ.

Soil carbon, nitrogen, and organic matter were significantly affected by variations in soil fissure status induced by mining disturbance (Table 1 and Supplementary Figure S2). In FCZ, TC, MBC, and DOC were significantly lower than those in FDZ (*p* < 0.05). Specifically, TC was 17.34 ± 1.44 g/kg in FCZ compared to 22.32 ± 1.13 g/kg in FDZ, MBC was 53.4 ± 4.84 mg/kg in FCZ versus 80.5 ± 9.76 mg/kg in FDZ, and DOC was 55.96 ± 2.81 mg/kg in FCZ compared to 67.51 ± 1.64 mg/kg in FDZ, indicating declines of 22.3, 33.7, and 17.1%, respectively. Furthermore, compared with the FDZ region, the contents of SOC (4.33 ± 0.59 g/kg and 4.46 ± 0.28 g/kg), Humus (1.01 ± 0.1 g/kg and 1.15 ± 0.06 g/kg), and HA (0.68 ± 0.08 g/kg and 0.85 ± 0.09 g/kg) decreased in FCZ, accounting for reductions of 2.9, 11.8, and 20.69%, respectively. Hm, the largest fraction of molecules remained stable in both sampling regions (3.31 ± 0.49 g/kg and 3.31 ± 0.24 g/kg). While most organic matter decreased in FCZ, FA increased from 0.29 ± 0.06 g/kg in FDZ to 0.33 ± 0.03 g/kg in FCZ, an increase of 14.27%. NO_3_-N, MBN, and AHN in FCZ also decreased significantly compared with those in FDZ. NO_3_-N content dropped from 4.65 ± 0.41 mg/kg in FDZ to 2.36 ± 0.28 mg/kg in FCZ, MBN from 8.76 ± 1.32 mg/kg to 5.55 ± 0.67 mg/kg, and AHN from 28.88 ± 1.33 mg/kg to 23.92 ± 1.84 mg/kg, representing reductions of 49.2, 36.6, and 17.3%, respectively. The contents of AK and AP in FCZ were 58.5 ± 5.0 mg/kg and 1.0 ± 0.2 mg/kg, whereas these were 72.7 ± 7.4 mg/kg and 1.3 ± 0.2 mg/kg in FDZ, representing decreases of 19.5 and 22.7%, respectively. Similarly, compared with FDZ, there was an overall reduction of 8.4% in TP and 0.4% in total potassium (TK) in FCZ. However, heavy metals had higher contents in FCZ compared to FDZ, including Cd (0.11 ± 0.02 mg/kg and 0.08 ± 0.02 mg/kg), Cr (20.62 ± 1.67 mg/kg and 17.35 ± 1.35 mg/kg), Fe (22.38 ± 1.51 g/kg and 20.12 ± 1.05 g/kg), and Mn (0.23 ± 0.02 g/kg and 0.23 ± 0.01 g/kg). These variables increased by 36.5% for Cd, 18.84% for Cr, 11.22 for Fe, and 6.18% for Mn. Conversely, only Cu (5.06 ± 0.55 mg/kg and 5.33 ± 0.45 mg/kg) and As (5.33 ± 0.67 mg/kg and 5.57 ± 0.38 mg/kg) in FCZ had lower contents than that of FDZ, the declines accounted for 5.06 and 4.33%, respectively. To sum up, soil nutrients decreased but metals generally increased from FDZ to FCZ.

**Figure 2:**
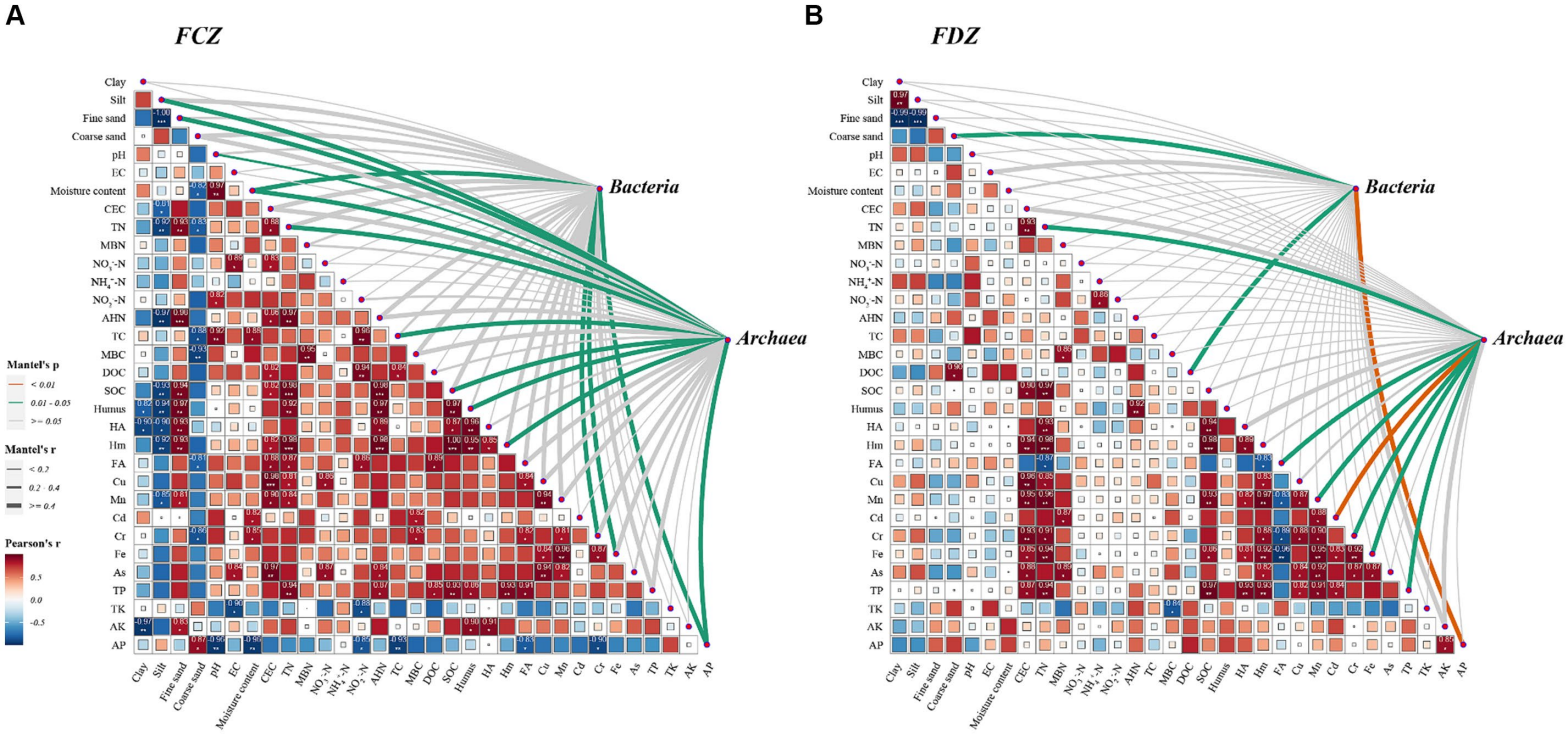
Pearson correlation matrix of the edaphic factors, and the correlation between microbial community composition and the edaphic factors using the Mantel test. **(A)** Pearson and Mantel results in the FCZ. **(B)** Pearson and Mantel results in the FDZ. ^*^p < 0.05, ^**^p < 0.01, and ^***^p < 0.001. The strength of the correlation is represented by the thickness of the lines, and the significance *p*-values are annotated with colors (yellow: *p* < 0.01; green: *p* < 0.05; gray: *p* > 0.05).

In the study area, the correlations between the edaphic factors in FCZ differed significantly from those in FDZ (Figures 2A,B). The heatmap results revealed that edaphic factors had high-level associations in FCZ and the coefficient was generally greater than that in FDZ. Only CEC and TN had significant positive correlations with heavy metals (such as Cu, Mn, Cr, Fe, and As), and the correlation between heavy metals strengthened in FDZ. The results clearly showed that the relationship among soil physicochemical properties significantly deteriorated during the development process of soil fissures in the study area. Notably, the size distribution of soil particles was significantly correlated with soil physicochemical components in FCZ (Figure 2A). Clay and silt, which are smaller diameter particles, had a negative correlation with organic components (including SOC, Humus, HA, and Hm), AHN, CEC, and Mn, while fine sand particles with larger diameter showed a significant positive correlation with above-mentioned factors. Coarse sand was not significantly correlated with these variables but was negatively correlated with SMC, TC, MBC, and FA. This is a solid dataset indicating that the soil physicochemical properties were significantly affected by the different fissure states caused by mining disturbance, resulting in significant divergence in their co-appearance and correlations.

### 3.2 Soil microbial composition variations under two stages of fissure conditions

According to the results of microbial high-throughput sequencing, a total of 486,595 bacterial sequences and 607,615 archaeal sequences were obtained, with the number of valid archaeal sequences surpassing that of bacterial sequences, indicating a substantial and non-neglectable archaea community harbored in the soils. This study evaluated the microbial community’s richness, diversity, and evenness using the microbial alpha diversity index (Table 2). The Sobs and Chao indexes represent the observed and theoretical values of microbial richness, which had an average value of 2,563 and 3,170 for the bacteria community and 254 and 366 for the archaea community, indicating a higher microbial richness level of the bacteria community than the archaea community. The Shannon and the Simpson indexes represent the diversity of microbial communities. The bacterial community showed higher diversity levels than the archaea community, as the average values were 6.5 and 0.0035 for the bacteria community and 2.1 and 0.17 for the archaea community. The evenness of the bacterial community ranged from 0.25 to 0.29, while that of the archaeal community ranged from 0.02 to 0.04, suggesting that the archaea community was less evenly distributed.

**Table 2:** The alpha diversity estimators from the soil bacteria and archaea communities.

SampleID	Coverage		Sobs		Chao		Shannon		Simpson		Heip	
	Bacteria	Archaea	Bacteria	Archaea	Bacteria	Archaea	Bacteria	Archaea	Bacteria	Archaea	Bacteria	Archaea
NTT01	98.5%	99.8%	2,393	209	2,926	272	6.40	1.89	0.005	0.247	0.25	0.03
NTT02	98.4%	99.8%	2,635	240	3,186	330	6.53	2.21	0.004	0.158	0.26	0.03
NTT03	98.0%	99.8%	2,633	271	3,279	424	6.56	2.11	0.003	0.165	0.27	0.03
NTT04	98.0%	99.8%	2,712	258	3,270	332	6.64	2.25	0.003	0.135	0.28	0.03
NTT05	97.7%	99.6%	2,579	246	3,272	447	6.56	2.20	0.004	0.142	0.27	0.03
NTT06	97.8%	99.8%	2,624	392	3,296	414	6.64	2.41	0.003	0.137	0.29	0.03
NTT07	98.2%	99.8%	2,630	214	3,070	282	6.61	2.25	0.003	0.144	0.28	0.04
NTT08	97.9%	99.8%	2,408	165	3,089	251	6.56	1.87	0.003	0.204	0.29	0.03
NTT09	97.2%	99.8%	2,465	243	3,268	350	6.57	2.04	0.003	0.169	0.29	0.03
NTT10	98.4%	99.8%	2,731	248	3,300	423	6.51	2.15	0.004	0.151	0.25	0.03
NTT11	98.0%	99.8%	2,488	286	3,077	406	6.51	2.01	0.004	0.173	0.27	0.02
NTT12	98.2%	99.7%	2,460	279	3,009	458	6.54	1.86	0.003	0.214	0.28	0.02

**Figure 3:**
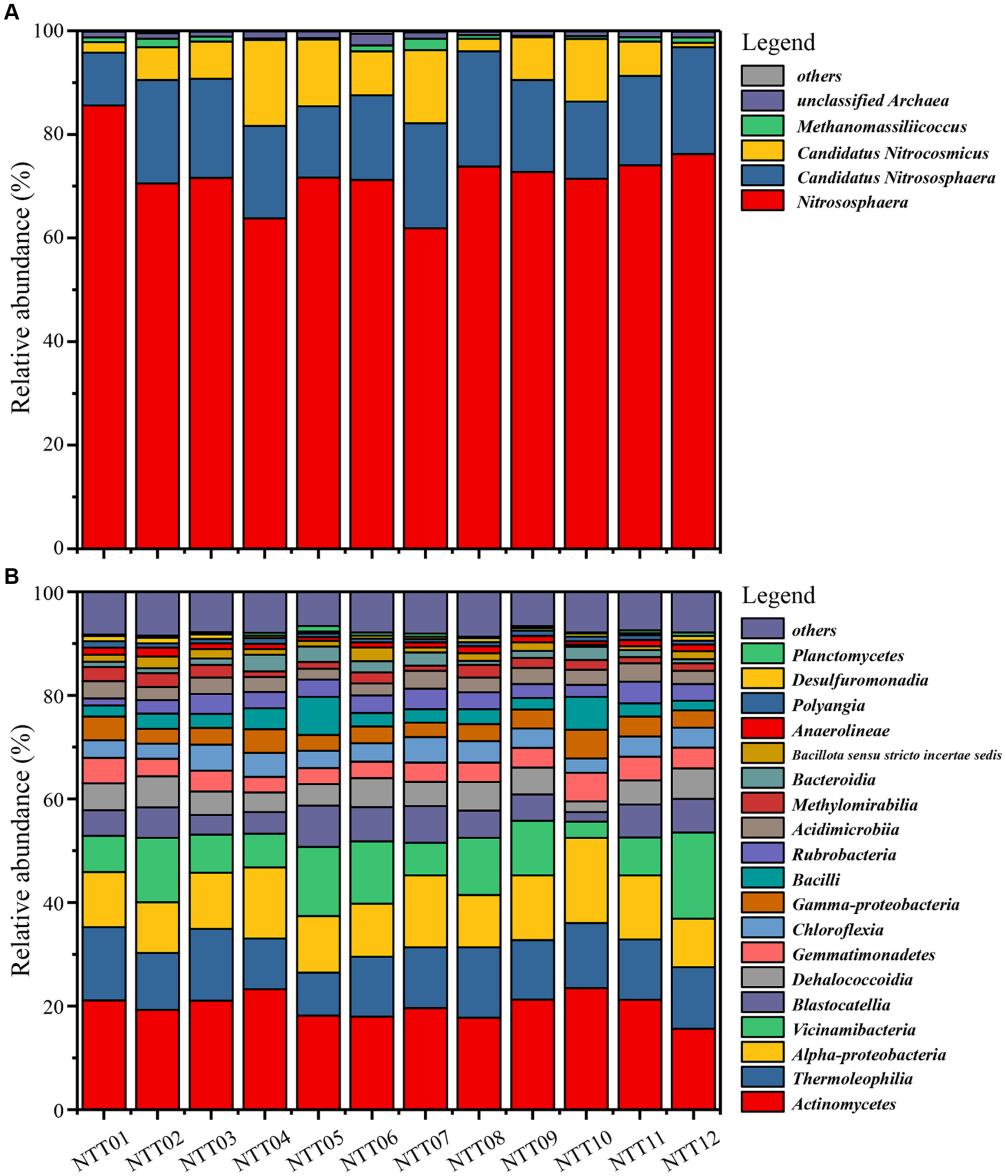
Microbial community compositions in the mining disturbance areas. **(A)** Archaea community at the genus taxonomic level. **(B)** Bacteria community at the class taxonomic level.

The archaea communities’ composition was simple and homogeneous (Figure 3A and Supplementary Table S1). *Nitrososphaeria* class, the most broadly distributed chemolithoautotrophy of Archaea and known for its capacity of aerobic oxidation of ammonia (Kerou et al., 2016), had an overwhelming abundance of more than 95% in all soil samples. At the genus taxonomic level, 72.04% ± 1.6% archaea lineages belonged to the genus *Nitrososphaera*, while 17.52% ± 0.9% archaea were identified as Candidatus *Nitrososphaera*, genetically similar to those of the *Nitrososphaera* genus. Additionally, 8.17% ± 1.4% of archaea belonged to an unclassified genus, Candidatus *Nitrocosmicus*. The aforementioned archaea showed no significant difference between the FDZ and FCZ groups (*p* > 0.05), indicating that mining disturbance has a relatively minor impact on the distribution of dominant archaea. Correspondingly, *Methanomassiliicoccus* (0.9% ± 0.2%) and numerous rare species (classified as “others” in this study) with relative abundances less than 1% showed significant differences between the two distinct sampling regions (*p* < 0.05), indicating mining disturbance has a greater impact on rare species within the archaea community.

The bacteria community was more complex and divergent compared to the archaea community. Due to many bacterial sequences not matching to the database at the genus taxonomic level, the bacteria composition was analyzed at the class taxonomic level (Figure 3B and Supplementary Table S2). No single bacterial lineage exceeded 25% relative abundance within the bacterial community. The most abundant bacteria, *Actinomycetes*, had an average abundance of 20.0% ± 0.6% across all soil samples, with small variations among sampling points. *Thermoleophilia*, *Alphaproteobacteria*, and *Vicinamibacteria* maintained relative abundances of around 10%, with average values of 11.79% ± 0.5, 11.75% ± 0.6, and 9.46% ± 1.1%. *Blastocatellia*, *Dehalococcoidia*, *Gemmatimonadetes*, *Chloroflexia*, *Gammaproteobacteria*, and *Bacilli* were all top 10 abundant bacteria, with abundances of 5.46% ± 0.5, 4.77% ± 0.3, 3.92% ± 0.2, 3.84% ± 0.2, 3.67% ± 0.2, and 3.36% ± 0.5%, respectively. Furthermore, *Rubrobacteria*, *Acidimicrobiia*, *Methylomirabilia*, *Bacteroidia*, *Bacillota sensu stricto incertae sedis*, and *Anaerolineae* exceeded 1% of bacteria community. Similar to archaeal community, there was no significant difference (*p* > 0.05) in the relative abundances of bacteria above 1% between FDZ and FCZ, while some rare species (e.g., *Rhodothermia*, no-ranked *Armatimonadota*, and no-ranked *Bacteroidota*) exhibited significant differences (*p* < 0.05) between the two different regions.

### 3.3 Microbial beta diversity characteristics under different soil fissure stages

Soil microorganisms play a vital role in maintaining the surface ecological environment and geochemical elements cycling, especially in ecologically vulnerable areas. Dry climate, scarce rainfall, and extensive coal mining activities in the study area destroyed the original structures and stresses of soil and rock and further exacerbated the vulnerability of the surface ecological environment. To quantitatively describe variations in microbial community compositions and how they respond to the mining activities, microbial beta-diversity analysis was employed. Microbial beta-diversity of the archaea and bacteria communities was analyzed by NMDS and PCoA methods, and both analyses were conducted at the same taxonomic level to ensure the consistency and comparability of the results.

**Figure 4:**
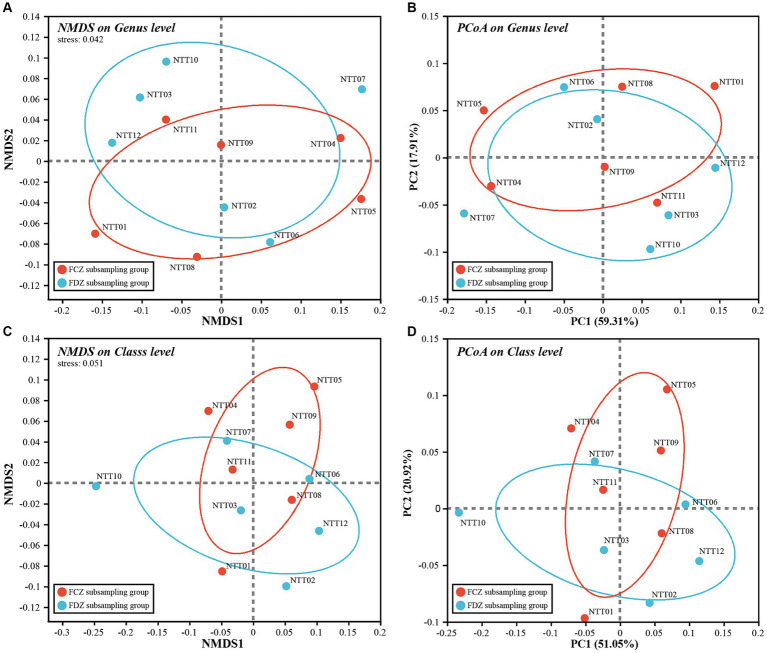
NMDS and PCoA biplot of **(A,B)** archaea community and **(C,D)** bacteria community, respectively.

Briefly, the stress values of NMDS results for the microbial communities were less than or close to 0.05, and the explanatory degree of the first two components (PC1 and PC2) in PCoA results exceeded 70%. This indicated high representativeness, allowing for a quantitative description of microbial composition characteristics (Figure 4). The distribution of the archaeal community was scattered with no obvious clustering between samples, primarily following the direction of the horizontal axis on the biplots (Figures 4A,B). Rare species could be the main attributions for the scattered pattern since significant variations observed in the rare species among the archaeal communities. Additionally, the archaeal communities collected from FDZ and FCZ overlapped on the biplot. The archaeal communities in FDZ showed more scattered distribution compared to that of FCZ on the plot, indicating more pronounced differences. Comparatively, the distribution of the bacterial community on the biplots appeared to be more clustered compared to the archaea community (Figures 4C,D), indicating a higher similarity in bacteria community compositions. In the biplot, samples from FDZ were generally distributed along the horizontal axis while those from FCZ were distributed along the vertical axis, hinting that the bacteria communities in FDZ and FCZ regions were influenced by distinct dominant factors.

Mining activities lead to significant changes in soil physicochemical properties due to soil fissure development and the subsequent closure. From the results of microbial beta diversity, the impact of mining disturbances on bacteria communities was more profound than that of archaea communities. Although the distribution characteristics of the two bacteria community subgroups on the biplot were different, the overall beta diversity differences between FCZ and FDZ were not significantly greater than within-group differences (ANOSIM, *p* > 0.05), reflecting the complexity of soil microbial communities.

### 3.4 Relationships between the edaphic factors and soil microbial community

In the present study, under the two different fissure statuses, the interactions between various environmental indicators and soil bacteria and archaea communities were determined by the Mantel test (Figure 2). Results showed that the bacteria community was significantly correlated with SMC, Cr, Fe, and AP, while the archaea community was strongly correlated with soil particle compositions (including silt and fine sand particles), pH, SMC, TN, TC, DOC, Hm, HA, Cr, Fe, and AP in the FCZ subregion (Figure 2A). In contrast, three environmental variables including coarse sand composition, DOC, and AP were significantly associated with the bacteria community in the FDZ region. At the same time, TN, FA, Mn, Cd, Cr, Fe, and TP likely impacted the archaea community due to significant correlations (Figure 2B). Archaea communities showed stronger interactions with environmental variables than bacteria communities under both stages of soil fissures, in terms of the number of associated environmental variables. This result indicated that the impact on archaeal communities may be more significant and far-reaching, despite variations in soil physicochemical properties caused by mining disturbances. Furthermore, for both bacterial and archaeal communities, the number and strength of significant correlations with environmental variables were fewer and weaker in the FDZ region than in the FCZ region, suggesting that the associations between microorganisms and soil environments deteriorated during the development of soil fissure, consistent with the observed deterioration in correlations among edaphic factors.

**Figure 5:**
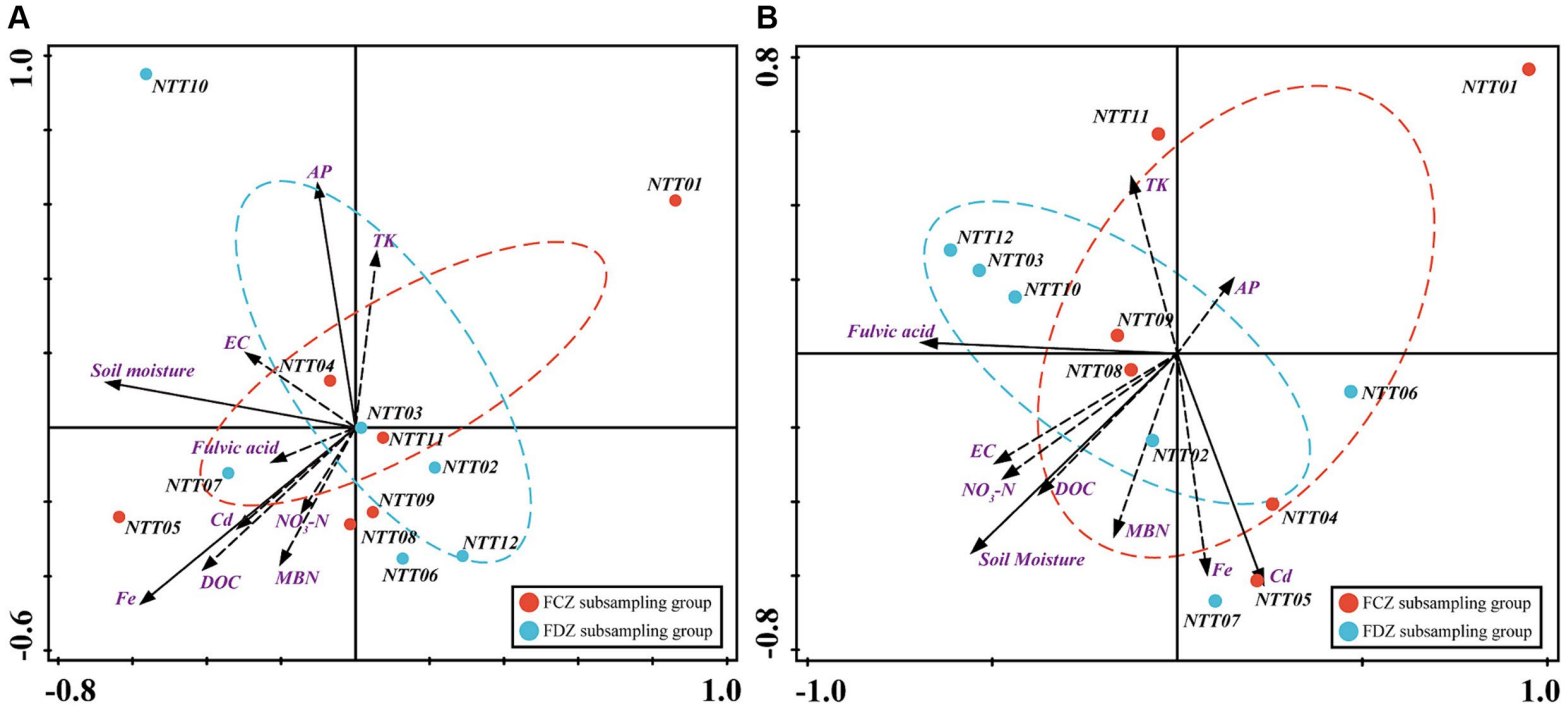
Drivers of microbial community composition. Redundancy analysis (RDA) between **(A)** bacteria community and **(B)** archaea community indicating the relationship between the edaphic factors and microbial communities at the OTU taxonomic level.

In this study, a total of 32 soil physicochemical variables were measured. However, collinearity among these parameters could obstruct the accuracy of analyses regarding the relationship between environmental conditions and soil microbial communities. To address this, a collinearity test was conducted, and 10 parameters were selected, including SMC, EC, MBN, NO_3_-N, DOC, FA, Cr, Fe, TK, and AP. The two-dimensional ordination plot generated by RDA results showed that the top two components accounted for more than 50% of the overall community variance (Figure 5). Edaphic factors had different impacts on bacteria and archaea communities in FCZ and FDZ. Fe, SMC, and AP had significant impacts on the bacteria community (*p* < 0.05), explaining total variances of 17.8, 17, and 12%, respectively (Figure 5A and Supplementary Table S3). MBN also accounted for a high level of explanatory degree (12.2%). In addition, the explanations of EC and FA were greater than 5%. In the FCZ subgroup, the edaphic factors, including Fe, Cd, MBN, FA, DOC, and NO_3_-N, showed strong influences on bacterial communities. Among these factors, Fe, DOC, and MBN exhibited greater contributions. In the FDZ subregion, the direction of SMC, EC, AP, and TK were the main influential factors. The influence of the physicochemical factors on the archaea community was also depicted by the RDA biplot (Figure 5B and Supplementary Table S4). The impact of SMC, FA, and Cd on the archaea community was significant (*p* < 0.05) and these three environmental factors collectively explained 53.8% of the overall community variations, with individual contributions of 25.6, 19.1, and 13.6%, respectively. Additionally, NO_3_-N, Fe, DOC, and EC accounted for 9.3, 8.9, 7.9, and 5.5% of the overall community variance. In the FCZ subsampling area, SMC, DOC, NO_3_-N, EC, AP, and MBN exhibited strong influences on the soil archaea communities, and SMC had the greatest contribution. In contrast, in the FDZ, FA, Cd, and Fe presented a high correlation with the archaea community, and FA and Cd significantly correlated with archaea communities (*p* < 0.05).

### 3.5 Impacts of fissure conditions on microbial co-occurrence network

**Figure 6:**
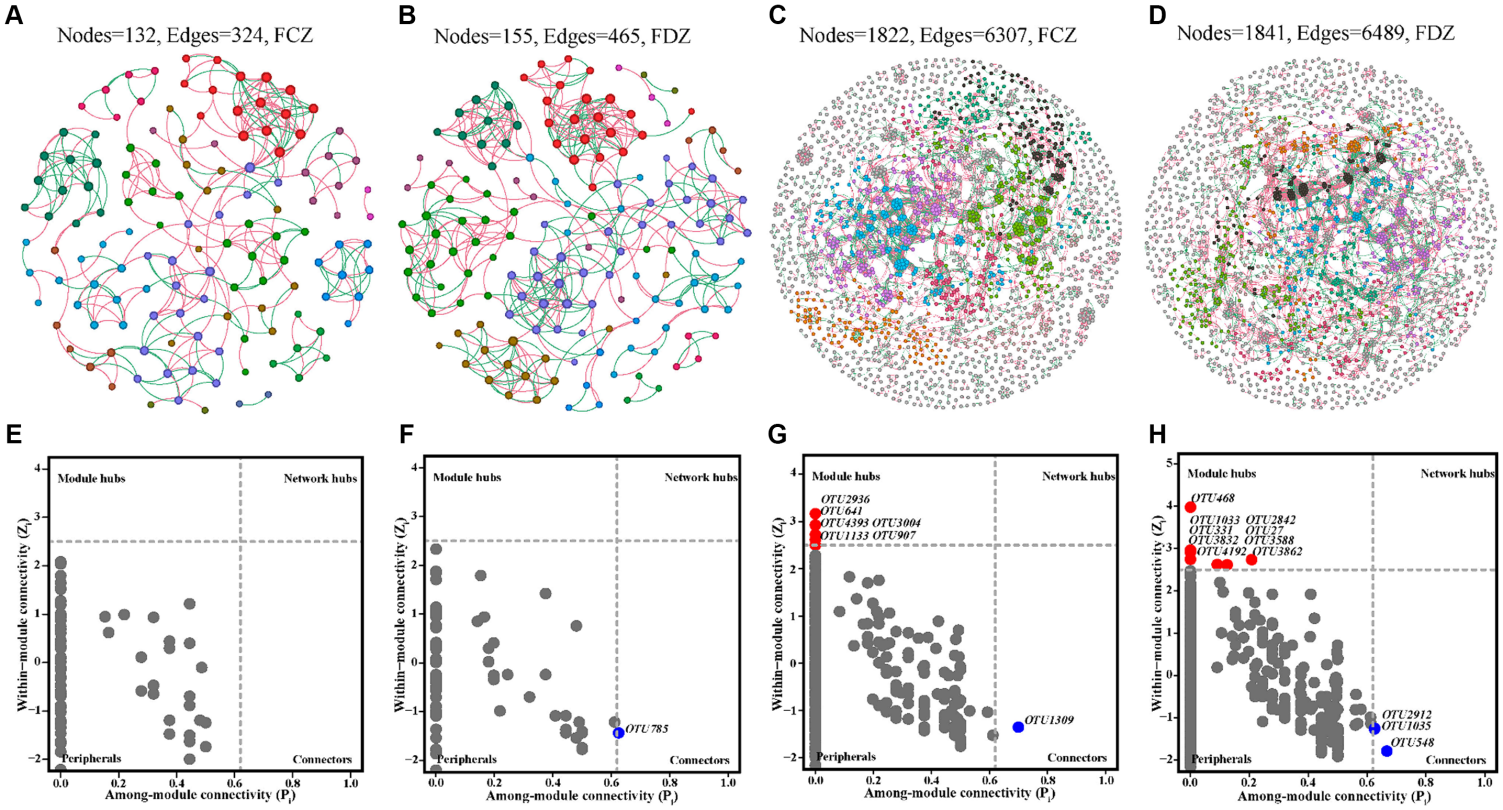
Microbial co-occurrence networks of archaea community in **(A)** FCZ, **(B)** FDZ, and bacteria community in **(C)** FCZ, **(D)** FDZ, respectively. The corresponding results about the roles of nodes in co-occurrence networks as indicated by their values of within-module connectivity (Zi) and among-module connectivity (Pi) of archaea community in **(E)** FCZ, **(F)** FDZ, and bacteria community in **(G)** FCZ, **(H)** FDZ.

Soil microbial co-occurrence networks were used to explore the interactions among soil microorganisms in the FCZ and FDZ subregions. No significant difference in the number of nodes, edges, and avgK of the bacterial community co-occurrence network was observed under the two different stages of soil fissure (Figures 6A–D and Table 3). Briefly, the number of microbial co-occurrence network nodes, edges and avgK of the bacteria communities in FCZ was 1,822, 6,307, and 6.923. Correspondingly, the number of nodes, edges, and avgK of the bacteria communities in FDZ was 1,841, 6,489, and 7.049. However, as for the archaeal communities, significant difference was observed. The microbial co-occurrence network of archaea communities in FDZ had more interactions than those in FCZ, in terms of the number of nodes (155 in FDZ, 132 in FCZ), edges (465 in FDZ, 324 in FCZ), and avgK (6 in FDZ, 4.909 in FCZ). In addition, soil microbial co-occurrence network in FDZ had closer interactions than FCZ based on the GD values (Bacteria: 14.053 in FDZ, 15.006 in FCZ; Archaea: 6.623 in FDZ, 7.394 in FCZ; Table 3). Stronger internal microbial interactions reflected the variations of the external soil environment. The correlation analysis and Mantel result both suggested that the associations among the edaphic factors deteriorated during the development of soil fissures, which may be the main reason for the changes in the microbial co-occurrence network.

**Table 3:** The microbial co-occurrence network topological parameters between the two soil fissure states.

Topological parameter	Bacteria		Archaea	
	FCZ	FDZ	FCZ	FDZ
Nodes	1,822	1,841	132	155
Edges	6,307	6,489	324	465
Average path distances (GD)	15.006	14.053	7.394	6.623
Positive edge ratios	56.48%	58.90%	59.26%	55.70%
Average degree (avgK)	6.923	7.049	4.909	6.000
Average clustering coefficient (avgCC)	0.537	0.529	0.569	0.591
Centralization of degree (CD)	0.012	0.016	0.054	0.045
Module	145	143	14	13
Modularity	0.847	0.836	0.796	0.776
Peripheral species	1,815	1,829	132	154
Connector hubs	1	3	0	1
Module hubs	6	9	0	0

The positive edge ratios in the co-occurrence networks of both bacterial and archaeal communities were relatively low, ranging from 55.7 to 59.26% in both FCZ and FDZ groups. This indicated interspecific competitive (or antagonistic) interactions were notable under the situation of soil disturbed by mining disturbances, regardless of whether the fissures were in development or developed state. According to Zi and Pi values, most nodes in the co-occurrence networks were peripheral (Figures 6E–H), and the number of modules of the bacterial communities (145 in FCZ, 143 in FDZ) was much higher than that of the archaeal communities (14 in FCZ, 13 in FDZ), suggesting greater abundance and complexity in bacterial communities. Soil bacteria networks had only 1 connector hub and 6 module hubs in FCZ, but those from FDZ had up to 3 connector hubs and 9 module hubs. The co-occurrence network of archaeal communities showed similar characteristics: archaeal networks had zero connector hubs or module hubs in FCZ, while the FDZ region had 1 connector hub but zero module hubs. These results indicated that soil microbial communities had more complex and interactive compositions under the soil fissures development states. Rare species constituted module hubs and connectors (Figures 6E–H), highlighting their keystone roles in microbial co-occurrence networks.

## 4 Discussion

### 4.1 Impacts of the fissure status on edaphic factors

Coal mining activities have changed the stability of geological structures, especially soil fissures that occurred in the topsoil layer (Liu et al., 2019; Yang et al., 2019). Generally speaking, soil fissures experience two distinct stages: fissure development and subsequent fissure closure (Kang et al., 2023). Previous studies have shown that mining activities disturbed soil physicochemical conditions and nutrient levels to varying degrees (Ma et al., 2019; Wang et al., 2021). Lots of effort paid attention to whether and to what extent mining disturbance changes soil conditions. However, little is known about whether the fissure statuses induced by mining activities affect the edaphic factors and how soil microbial communities respond to these fissure development states. Here, a shallow buried coal mining area was selected to investigate whether the fissure status impacts soil physicochemical characteristics. In this area, according to the accurate locations of active mining panels and surface soil conditions, two states of soil fissures were identified. Almost all organic matters (e.g., MBC, MBN, DOC, AHN, and HA), SMC, AK, AP, pH, and CEC, had lower contents in the soil fissure closure zone (FCZ). This result differed from some previous studies showing that the nutrients were depleted upon fissure development (Ma et al., 2019; Wang et al., 2021). In addition, heavy metals and fine soil particles including clay and silt were enriched in FCZ (Table 1). The increase of soil fissures altered the size distribution of soil particles during soil fissure development. Clay and silt, the smaller particles in soil compositions, had lower contents in FDZ (Table 1), which might be the result of the enhanced small-sized particle’s downward movement under the increase of soil fissure space and connectivity (Wang et al., 2021).

The expansion of soil fissures would reduce the tortuosity of the water flow path to promote vertical movement of the water and result in increasing infiltration (Karacan and Goodman, 2009; Bi et al., 2014), leading to an enhancement of the effective downward flow and increase in soil moisture content (Zang et al., 2012; Wang J. et al., 2017). The contents of soil physicochemical properties, except for heavy metals, increased to a certain extent with the increasing soil moisture content in FDZ (Table 1), due to the enhanced dissolution of soluble substances in topsoil and accumulated in the subsoil (Zhu et al., 2020). In addition, lower contents of heavy metals (e.g., Mn, Cd, Cr, and Fe) in FDZ were probably due to high adsorption or binding capacity with dissolved organic matter (Ponizovsky et al., 2006; Liu et al., 2021) and microbial weathering or reductive dissolution of metals into the mobilized forms (Sheng et al., 2021a, 2023a).

However, despite these increased nutrients in FDZ, the connections among soil physicochemical properties in this area were damaged by the process of the fissure development, weakening the relationships among factors (Wang et al., 2021; Zhang et al., 2022). Soil physicochemical variables in FCZ had high-level correlations, with greater coefficients than FDZ. The size distribution of soil particles strengthened in correlations with soil physicochemical components in FCZ, especially having a significant relationship with organic matters (such as AHN, HA, and Humus). This phenomenon hinted that the associations of soil physicochemical properties were recovered as the process of the soil fissures closed. This result was similar to the previous research illustrating that mining disturbance significantly reduced TN, NO_3_-N, and TC contents in the soil, and the correlations of soil variables were generally recovered after the mining activities ceased (Ngugi et al., 2018).

### 4.2 Soil fissure status affects microbial diversity, composition, and their co-occurrence

Soil nutrients, SMC, and inorganic properties were significantly altered in the mining disturbance area, leading to a large differentiation of soil microbial biomass, community structure, and their activities (Huang et al., 2015; Guo et al., 2021; Sheng et al., 2022; Sheng et al., 2023b). The dominant viewpoint held that surface subsidence reduced microbial richness and diversity of the soil microbial community. For instance, the relative abundance of predominant microbial lineages, such as *Pseudomonas*, *Gemmatimonas*, *Arthrobacter*, *Aciditerrimonas*, *Gaiella*, and *Sphingomonas* in the mining area decreased significantly (Shi et al., 2017; Wang et al., 2023). In addition, mining disturbance changed the soil microbial co-occurrence network, and the phylogenetic community assembly mechanism differed between mining-disturbed and non-disturbed areas (Ma et al., 2023; Wang et al., 2023). However, whether and how the soil microbial community’s diversity, composition, and interaction changed under the different soil fissure states induced by mining disturbance still lacked evidence and remained unclear.

In this research, we investigated soil microbial communities under the two states of soil fissure. The archaeal community was solely composed of *Nitrososphaeria* which had a predominant abundance exceeding 95% in FCZ and FDZ. For the bacterial community, *Actinomycetes*, *Thermoleophilia*, *Alphaproteobacteria*, and *Vicinamibacteria* were dominant bacteria members with an average abundance exceeding 10% across all soil samples, with some variations between FCZ and FDZ (Figure 3 and Supplementary Tables S1, S2). Although there were little variations from the perspective of dominant microbial community composition, this study reported a distinct composition of rare species (e.g., *Methanomassiliicoccus*, *Rhodothermia*, *Armatimonadota*) between FCZ and FDZ, as well as the microbial co-occurrence network were connected by rare species (Figure 6).

Microbial communities had close correlations with soil environment conditions, including different fissure states induced by mining disturbance (Poncelet et al., 2014; Shi et al., 2017). The distribution of microbial communities in FDZ and FCZ regions showed independent vertical characteristics, revealing that the edaphic factors had varying degrees of impact on bacterial and archaeal communities in soils at two different stages of soil fissures (Figures 4, 5). During the soil fissure development process in the present study, the original porosity and structure were destroyed and the relatively close correlations among soil edaphic factors were weakened, except for heavy metals. As a result, the archaeal community showed a high correlation with organic matters in the FCZ area, and transformed to the correlations with heavy metals in the FDZ region (Figure 5B). The bacteria community showed similar features and had much lower correlations with soil physicochemical conditions in the FDZ area (Figure 5A). When the external environment changes, microorganisms tend to strengthen internal connections in response to environmental variations (Lu et al., 2022). The soil microbial co-occurrence network depicted a more complex and interactive microbial network in the FDZ region, with the soil bacterial co-occurrence network having three connector hubs and nine module hubs in FDZ and lowered to one connector hub and six module hubs in FCZ. Similar characteristics were also observed in the archaeal network, which had one connector hub in FDZ but none in FCZ (Table 3). The results showed that the relationship between soil microorganisms and the environment is destroyed in the process of developing fissures.

Not only was the microbial community affected by mining activities, but the soil microbial diversity and biomass of the restored mining area also significantly differed from that in the undisturbed area (de Quadros et al., 2016). Over time, the bacterial communities on restored sites became progressively more similar to those of nonmined analog sites (Ngugi et al., 2018). However, some findings also argued that the microbial composition and abundance of the restoration site still had significant differences with the undisturbed area after a long period of restoration time (de Quadros et al., 2016; Du et al., 2021). In this research, the process of soil fissure closure was similar to the restoration sites (Ngugi et al., 2018). As the soil fissure closed, the disturbed correlations among soil physicochemical variables reconnected, showing higher and more significant correlations compared to those in FDZ (Figure 2). Correspondingly, the bacterial and archaeal communities in FCZ showed closer correlations with soil environmental parameters compared with the microbial communities in FDZ, both in terms of the number of significantly correlated variables and the value of their correlation coefficient (Figure 2). The compositions and variations of soil microbial community and microbial-soil interactions were largely attributed to the continuity of the soil environment, as well as the correlations among physicochemical variables (e.g., soil organic matter), which is conducive to the establishment of stable microbial communities (Guo et al., 2021; Luo et al., 2019).

### 4.3 Rare species of soil microbial community are vulnerable to mining disturbance

It is generally believed that when the environment changes, the main manifestations of microbial community variation are changes in dominant species or functional genes, especially in environments such as soil and groundwater. Soil edaphic factors, such as pH, salinity, SMC, organic matter, etc., showed high attributes in shaping microbial diversity, taxonomic composition, and functional groups (Ezeokoli et al., 2019; Guo et al., 2021; Xiao et al., 2021a). In consideration of soil fissure stages in mining disturbance areas, in the present study, we observed a significant variation occurring in rare species, instead of dominant microorganisms as the previous research reported (Xiao et al., 2021a). Archaeal lineage (e.g., *Methanomassiliicoccus*) and bacterial members (e.g., *Rhodothermia*, *Armatimonadota*) were significantly different between FCZ and FDZ (*p* < 0.05). Rare species with an abundance of less than 1% accounted for the main inter-group differences, indicating that mining disturbances have a greater impact on rare species. Previous studies have illustrated that rare species play an over-proportional role and might be a hidden driver in biogeochemical cycles (Jousset et al., 2017). Both common and rare species could contribute to soil ecological function. Dominant species make persistent, unique contributions to functional diversity, while rare species play key roles in changing environments (Chapman et al., 2018; Liang et al., 2020). Thus, it is vital to account for the abundance and diversity of rare species when interpreting their contributions to the entire microbial community diversity and variation in different soil fissure states.

**Figure 7:**
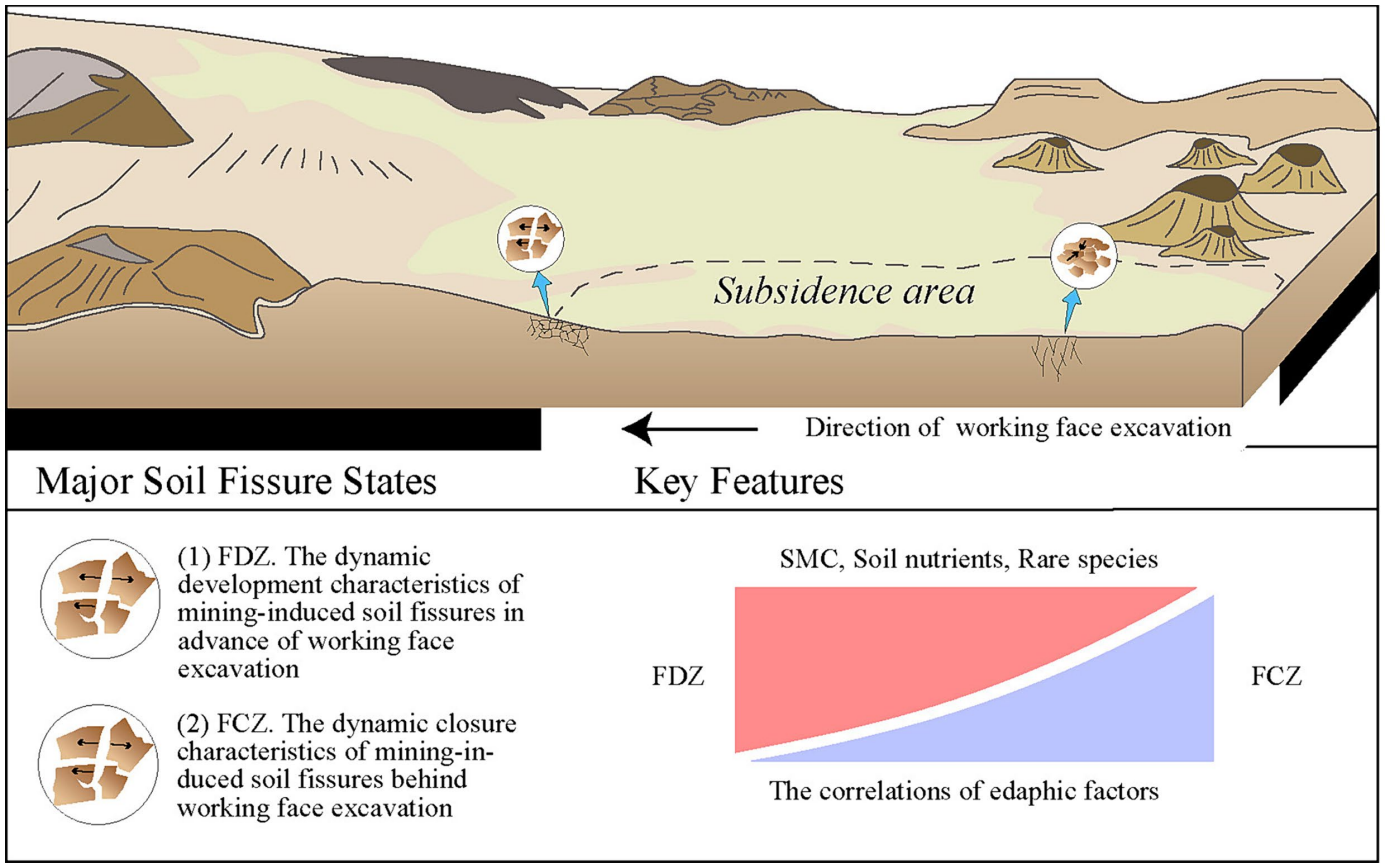
Conceptual map of the impacts of mining disturbance on soil edaphic factors and microbial community. Two different soil fissure states induced by mining activities significantly impacted the edaphic factors and the microbial community, especially in rare species.

The microbial co-occurrence network proved that rare species occupied an important soil ecological position in the study area, as the module hubs and connectors were composed of rare species rather than common species. In addition, rare species reinforced the overall interconnections of the soil microbial community. According to the compositions of connector hubs or module hubs, rare species such as *Nordella*, unclassified species of *Chloroflexia*, and *Acidimicrobiia* were connector hubs in microbial co-occurrence networks. As for module hubs, they were largely composed of rare species *Sphingomonas*, *Massilia*, *Candidatus Alysiosphaera*, *Rubritepida*, and other unclassified rare lineages (Figures 6E–H). These rare species connected microbial symbionts and provided habitat complexity in the view of co-occurrence relationships. Abundant taxa have been reported more sensitive to ecological disturbance situations, showing significant variation between non-disturbed and disturbed areas (Yuan et al., 2022). Two different soil fissure states induced by mining activities significantly impacted the edaphic factors and the microbial community (Figure 7). Compared with abundant species, rare species of soil microbial community were vulnerable to mining disturbance, as illustrated by microbial composition and co-occurrence network. Soil fissure states had a great impact on the soil edaphic factors and deteriorated the correlation between these variables in the process of soil fissure development, except for the correlation between heavy metals (Figure 6). Among these factors, soil moisture content was the most influential edaphic factor attributed to microbial variations (Figure 5). Controlled by the distinct states of soil fissures, the edaphic conditions showed key attributions to microbial communities, particularly affecting the abundance and ecological roles of rare species (Xiao et al., 2021b).

## 5 Conclusion

The effects of soil fissure status on the microbial community have been thoroughly investigated in a representative mining disturbance area. Here, we presented a novel insight into soil microbial community characteristics and variations under distinct soil fissure states through multiple statistical and bioinformatic approaches. Distinct soil fissure states significantly impacted the edaphic factors, especially in soil moisture content and organic matter. Soil microbial communities showed different structures under the two different fissure states, and the soil moisture content, pH, particle compositions, organic matter, and heavy metals contributed to the variation of microbial communities. Rare species of soil microbial community were vulnerable to mining disturbance and played keystone roles in the soil microbial community (e.g., *Nordella*, *Sphingomonas*, *Massilia*, and *Rubritepida*). Our work highlighted the impacts of soil fissure states on soil physicochemical properties and microbial communities, and expanded our knowledge of the effects of mining activities on the edaphic factors and microbial communities. In addition, future works should pay more attention to microbial function and metabolism (e.g., metagenomics, metatranscriptomics) under mining-disturbed conditions.

## Data Availability

The original contributions presented in the study are included in the article/Supplementary material, further inquiries can be directed to the corresponding authors.

## Footnotes

^1^https://blast.ncbi.nlm.nih.gov/Blast.cgi
