# Supplementary material for: Impact of soil fissure status on microbial community in mining-disturbed area, the northern Shaanxi province
Source: Front Microbiol. 2024 Aug 29;15:1463665. doi: 10.3389/fmicb.2024.1463665 (PMC11390389; doi:10.3389/fmicb.2024.1463665)
Supplement: Supplementary file 1 [file Data_Sheet_1.DOCX]

**SUPPLEMENTARY INFORMATION**

**Impact of soil fissure status on microbial community in mining-disturbed area: a case study from the northern Shaanxi Province**

Liang Guo^a^, Xianglong Chen^b^, Yizhi Sheng^b^, Nuan Yang^a,^*, Enke Hou^a,^*, Haisong Fang^a^

^a^ College of Geology and Environment, Xi’an University of Science and Technology, Xi’an, 710054, PR China

^b^ State Key Laboratory of Biogeology and Environmental Geology & MOE Key Laboratory of Groundwater Circulation and Environment Evolution, China University of Geosciences, Beijing 100083, PR China

*** Corresponding author:**

Nuan Yang

College of Geology and Environment, Xi’an University of Science and Technology, Xi’an, 710054, PR China

Email: [yangnuan@xust.edu.cn](mailto:yangnuan@xust.edu.cn)

Enke Hou

College of Geology and Environment, Xi’an University of Science and Technology, Xi’an, 710054, PR China

Email: [houek@xust.edu.cn](mailto:houek@xust.edu.cn)

2 supplementary Figure

4 supplementary Tables


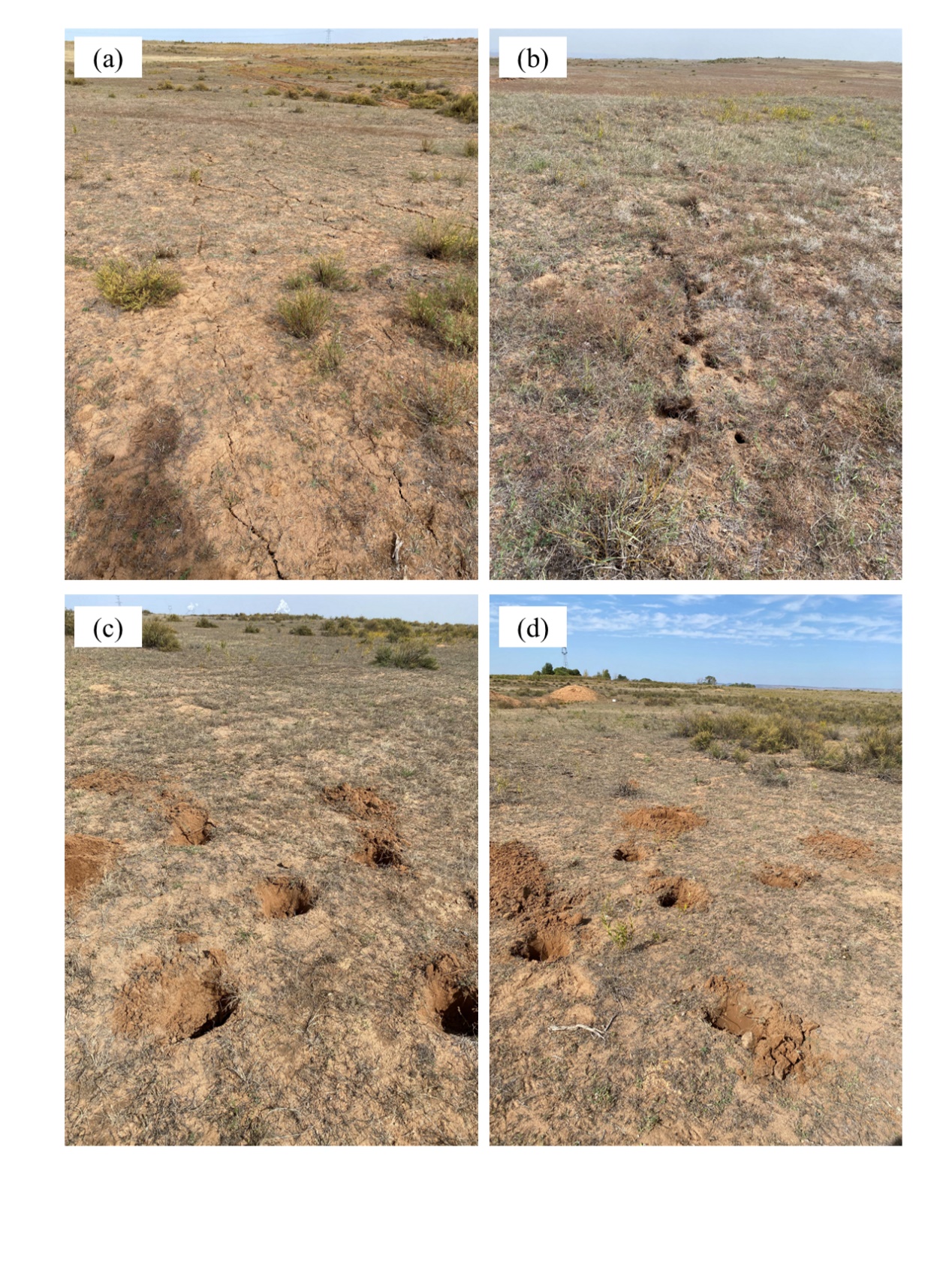


Figure S1. Representative sampling site images which were photoed by Guo Liang during field investigation. Images (a) and (b) were taken from soil fissure development zone (FDZ) and the fissures were clearly and obvious, while (c) and (d) were filmed from soil fissure closure zone (FCZ).


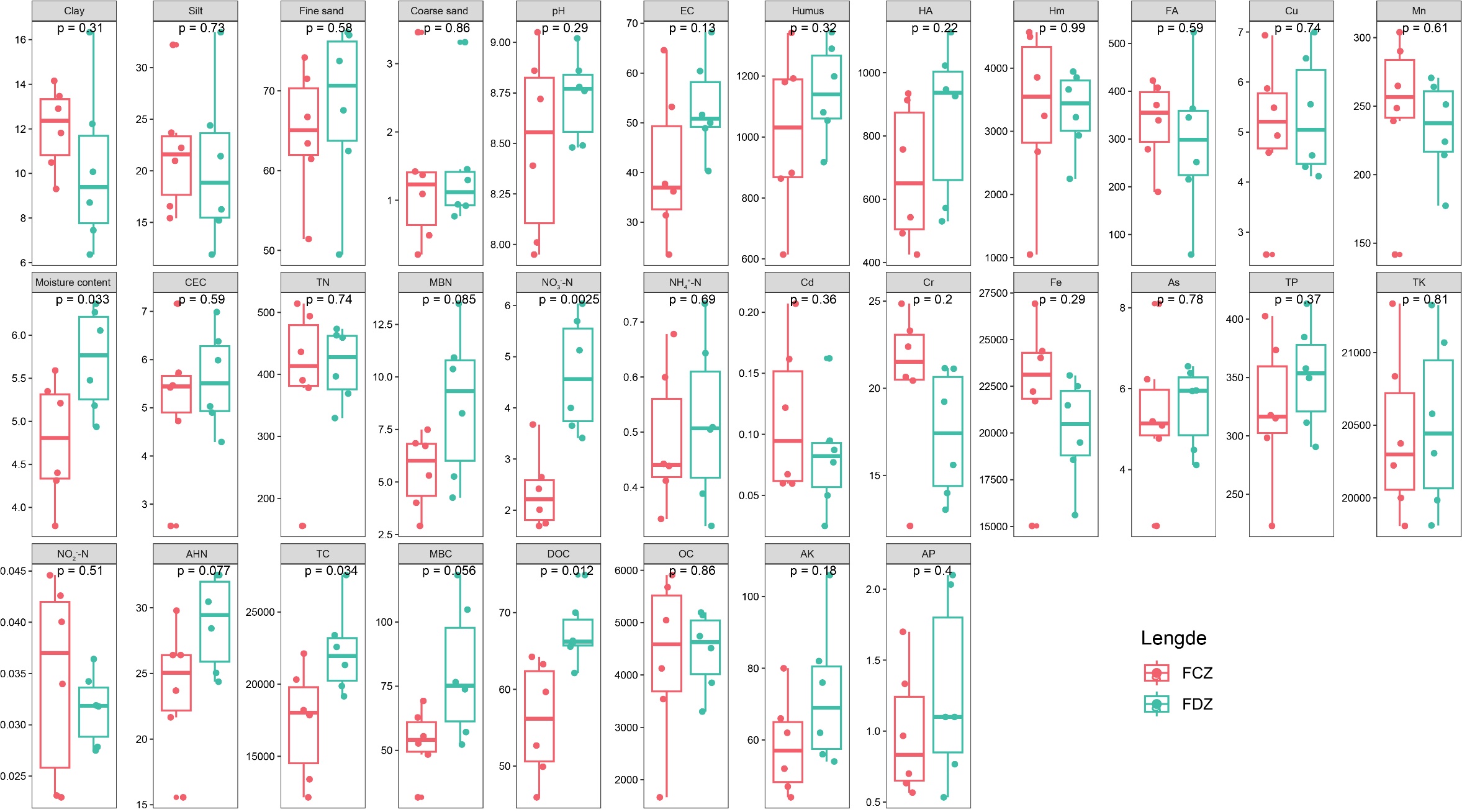


Figure S2. Box and whisker plot with significant test of the edaphic factors between the FCZ and FDZ subsampling area.

Table S1. Soil archaea communities’ compositions at the Genus taxonomic level.

| Taxonomy | NTT01 | NTT02 | NTT03 | NTT04 | NTT05 | NTT06 | NTT07 | NTT08 | NTT09 | NTT10 | NTT11 | NTT12 |
| --- | --- | --- | --- | --- | --- | --- | --- | --- | --- | --- | --- | --- |
| Nitrososphaera | 85.55% | 70.54% | 71.59% | 63.81% | 71.68% | 71.23% | 61.86% | 73.81% | 72.71% | 71.44% | 74.04% | 76.17% |
| Candidatus_Nitrososphaera | 10.21% | 19.96% | 19.15% | 17.82% | 13.73% | 16.32% | 20.28% | 22.23% | 17.80% | 14.86% | 17.24% | 20.68% |
| Candidatus_Nitrocosmicus | 2.05% | 6.35% | 7.16% | 16.65% | 12.87% | 8.49% | 14.14% | 2.46% | 8.23% | 12.15% | 6.61% | 0.85% |
| Methanomassiliicoccus | 0.92% | 1.60% | 0.96% | 0.28% | 0.32% | 1.15% | 2.19% | 0.66% | 0.30% | 0.54% | 0.84% | 1.02% |
| Unclassified Archaea | 1.13% | 1.11% | 0.95% | 1.34% | 1.27% | 2.24% | 1.22% | 0.67% | 0.90% | 0.89% | 1.19% | 1.13% |
| others | 0.15% | 0.45% | 0.19% | 0.11% | 0.14% | 0.56% | 0.31% | 0.17% | 0.06% | 0.13% | 0.07% | 0.15% |

Table S2. Soil bacteria communities’ compositions at the Class taxonomic level.

| Taxonomy | NTT01 | NTT02 | NTT03 | NTT04 | NTT05 | NTT06 | NTT07 | NTT08 | NTT09 | NTT10 | NTT11 | NTT12 |
| --- | --- | --- | --- | --- | --- | --- | --- | --- | --- | --- | --- | --- |
| Actinomycetes | 21.09% | 19.28% | 21.02% | 23.26% | 18.16% | 17.98% | 19.63% | 17.77% | 21.27% | 23.47% | 21.20% | 15.64% |
| Thermoleophilia | 14.13% | 10.98% | 13.86% | 9.78% | 8.31% | 11.54% | 11.71% | 13.56% | 11.47% | 12.58% | 11.65% | 11.86% |
| Alphaproteobacteria | 10.65% | 9.81% | 10.88% | 13.72% | 10.90% | 10.23% | 13.93% | 10.12% | 12.52% | 16.45% | 12.42% | 9.35% |
| Vicinamibacteria | 7.04% | 12.44% | 7.34% | 6.53% | 13.38% | 12.05% | 6.23% | 11.01% | 10.48% | 3.13% | 7.26% | 16.65% |
| Blastocatellia | 4.90% | 5.85% | 3.79% | 4.20% | 7.93% | 6.64% | 7.10% | 5.28% | 5.10% | 1.82% | 6.39% | 6.53% |
| Dehalococcoidia | 5.17% | 6.03% | 4.52% | 3.76% | 4.14% | 5.59% | 4.68% | 5.47% | 5.24% | 2.09% | 4.65% | 5.87% |
| Gemmatimonadetes | 4.94% | 3.36% | 4.03% | 2.99% | 3.14% | 3.15% | 3.77% | 3.81% | 3.77% | 5.54% | 4.58% | 3.98% |
| Chloroflexia | 3.37% | 2.90% | 5.02% | 4.63% | 3.34% | 3.62% | 4.88% | 4.10% | 3.75% | 2.75% | 3.89% | 3.88% |
| Gammaproteobacteria | 4.62% | 2.87% | 3.25% | 4.58% | 3.03% | 3.17% | 2.80% | 3.31% | 3.63% | 5.56% | 3.88% | 3.32% |
| Bacilli | 2.16% | 2.97% | 2.70% | 4.03% | 7.35% | 2.63% | 2.59% | 2.89% | 2.23% | 6.33% | 2.55% | 1.88% |
| Rubrobacteria | 1.31% | 2.58% | 3.86% | 3.17% | 3.38% | 3.38% | 3.97% | 3.27% | 2.70% | 2.30% | 4.20% | 3.18% |
| Acidimicrobiia | 3.36% | 2.51% | 3.18% | 2.87% | 2.09% | 2.33% | 3.45% | 2.83% | 3.09% | 2.94% | 3.52% | 2.56% |
| Methylomirabilia | 2.71% | 2.67% | 2.43% | 1.12% | 1.30% | 2.14% | 1.01% | 2.47% | 1.96% | 1.88% | 1.24% | 1.49% |
| Bacteroidia | 1.01% | 0.99% | 1.23% | 3.22% | 2.99% | 2.19% | 2.54% | 0.76% | 1.36% | 2.52% | 1.33% | 0.76% |
| Bacillota sensu stricto incertae sedis | 1.37% | 2.26% | 1.83% | 1.08% | 1.04% | 2.62% | 0.96% | 1.46% | 1.62% | 0.40% | 0.77% | 1.53% |
| Anaerolineae | 1.36% | 1.71% | 1.11% | 0.99% | 0.77% | 0.96% | 0.97% | 1.35% | 1.23% | 0.67% | 1.15% | 1.33% |
| Polyangia | 1.26% | 0.80% | 0.88% | 1.16% | 0.67% | 0.77% | 0.66% | 0.81% | 1.03% | 0.96% | 0.96% | 0.71% |
| Desulfuromonadia | 0.99% | 1.10% | 0.91% | 0.42% | 0.36% | 0.57% | 0.45% | 0.76% | 0.52% | 0.56% | 0.37% | 0.89% |
| Planctomycetes | 0.29% | 0.38% | 0.35% | 0.60% | 1.08% | 0.60% | 0.60% | 0.35% | 0.37% | 0.19% | 0.52% | 0.69% |
| others | 8.28% | 8.49% | 7.83% | 7.89% | 6.64% | 7.86% | 8.07% | 8.63% | 6.65% | 7.87% | 7.48% | 7.88% |

Table S3. Significance test of the edaphic variables and bacteria communities (referring to OTU taxonomic level)

| Variables | Explains (%) | Contribution (%) | pseudo-F | P |
| --- | --- | --- | --- | --- |
| Iron | 17.8 | 20 | 2.2 | 0.034 |
| Moisture content | 17 | 19 | 2.3 | 0.016 |
| Available phosphorus | 12 | 13.5 | 2 | 0.038 |
| Microbial biomass nitrogen | 12.2 | 13.7 | 1.8 | 0.066 |
| Fulvic acid | 6 | 6.8 | 1 | 0.354 |
| Total potassium | 4.8 | 5.4 | 0.8 | 0.58 |
| Organic carbon | 4.7 | 5.3 | 0.7 | 0.638 |
| Nitrate nitrogen | 4.5 | 5.1 | 0.7 | 0.638 |
| Electrical conductivity | 7.3 | 8.2 | 1.1 | 0.418 |
| Cadmium | 2.7 | 3 | 0.2 | 0.786 |

Table S4. Significance test of the edaphic variables and archaea communities (referring to OTU taxonomic level)

| Variables | Explains (%) | Contribution (%) | pseudo-F | P |
| --- | --- | --- | --- | --- |
| Moisture content | 25.6 | 27.2 | 3.4 | 0.032 |
| Fulvic acid | 19.1 | 20.3 | 3.1 | 0.036 |
| Cadmium | 13.6 | 14.4 | 2.6 | 0.05 |
| Nitrate nitrogen | 9.3 | 9.8 | 3 | 0.06 |
| Iron | 8.9 | 9.4 | 1.9 | 0.132 |
| Organic carbon | 7.9 | 8.4 | 1.9 | 0.154 |
| Electrical conductivity | 5.5 | 5.8 | 2.2 | 0.134 |
| Available phosphorus | 2.6 | 2.8 | 1 | 0.428 |
| Total potassium | 1 | 1.1 | 0.3 | 0.79 |
| Microbial biomass nitrogen | 0.7 | 0.8 | 0.1 | 0.85 |
